# Overexpression of RNA m^6^A demethylase FTO enhances the yield of natural rubber in lettuce

**DOI:** 10.1186/s43897-025-00190-y

**Published:** 2026-02-06

**Authors:** Bin Hu, Na Yang, Wei Li, Zemin Zheng, Yuan Lin, Kun Wang, Juan Chen, Zaihui Zhou, Yunxia Qin, Suhan Qiu, Haitao Huang, Jiahui Li, Xiangyu Long

**Affiliations:** 1 State Key Laboratory of Tropical Crop Breeding/Key Laboratory of Biology and Genetic Resources of Rubber Tree, Rubber Research Institute, Sanya Research Institute of Chinese Academy of Tropical Agricultural Sciences, Sanya/Haikou Hainan, 572024/571101 China; 2 Mianyang Academy of Agricultural Sciences, Mianyang, 621023 P.R. China; 3 College of Life Sciences and Medicine, Key Laboratory of Plant Secondary Metabolism and Regulation in Zhejiang Province, Zhejiang Sci-Tech University, Hangzhou, 310018 China; 4 National Key Laboratory of Crop Genetic Improvement and National Center of Plant Gene Research (Wuhan), Huazhong Agricultural University, Wuhan, 430070 P.R. China

Natural rubber (NR), with its wide range of industrial applications, is extensively utilized in various commercial activities. Despite more than 2,500 plant species being capable of producing NR, the primary commercial source of NR remains the rubber tree, *Hevea brasiliensis* (Mooibroek and Cornish 2000). Recently, some rubber-producing plants with commercial potential, such as lettuce and the Russian dandelion, *Taraxacum kok-saghyz*, have garnered the interest of researchers (Cao et al. 2025; Cherian et al. 2019). *FTO*, as a conserved demethylase, was involved in glycolysis metabolism (Wang et al. 2023), gluconeogenesis (Peng et al. 2019) and cell development and differentiation (Deng et al. 2021). Overexpression of FTO in various crops enhances yield (Yu et al. 2021), but whether m^6^A modification affects rubber biosynthesis in rubber-producing plants remains unknown.

In this study, we constructed an FTO overexpression vector (Fig. S1A) and transformed it into lettuce (Ninja) for phenotypic observation. Through copy number analysis of FTO-transgenic T0 plants, single-copy transgenic lines were selected for subsequent phenotypic evaluation in the T2 generation (Fig. S1B). The needle puncture test showed that the FTO-OE lines exuded more latex in the stems (Fig. S1C) and leaves (Fig. S1D) compared to the WT, and the chlorophyll content (Fig. S1E) and stem girth (Fig. S1F) were significantly increased in the FTO-OE lines. We demonstrate that the overexpression of the RNA demethylase FTO (Fig. 1A-C) in lettuce led to an increase in latex yield ranging from 6.89 to 14.21% (Fig. 1D, E). We aimed to determine if there were changes in laticifer cells in FTO-OE plants by employing iodine-bromine solution and Sudan Black B staining techniques on stems, roots and leaves (Castelblanque et al. 2018); Chao et al. 2023); Zhang et al. 2015). The results showed a significant increase, ranging from 54.65 to 460.12%, in the relative areas of laticifer cells stained with iodine-bromine in roots (Fig. 1F, H) and stems (Fig. 1G, I). Additionally, Sudan Black B staining on leaves indicated more intense staining of laticifer cells in the OE-19 (Fig. S1G). Further research revealed that molecular weight (Fig. 1J) and PDI (Fig. S1H) did not change in all OE lines as determined by high-performance liquid chromatography on a gel-permeation column (HPLC-GPC) (Fig. S1I). Using 1,4-dioxane as a standard, the rubber hydrocarbon content in the dry latex of transgenic lines was quantitatively determined by nuclear magnetic resonance (NMR). The NMR results showed that the rubber hydrocarbon content in the dry latex of the FTO-OE lines exhibited an increase ranging from 40.37 to 802.57% (Fig. 1K, L). While, the FTO-OE lines exhibited earlier flowering by 8–20 days, which is advantageous for latex harvesting in lettuce as latex content peaks during bolting and flowering (Fig. S1J, K).

**Fig. 1:**
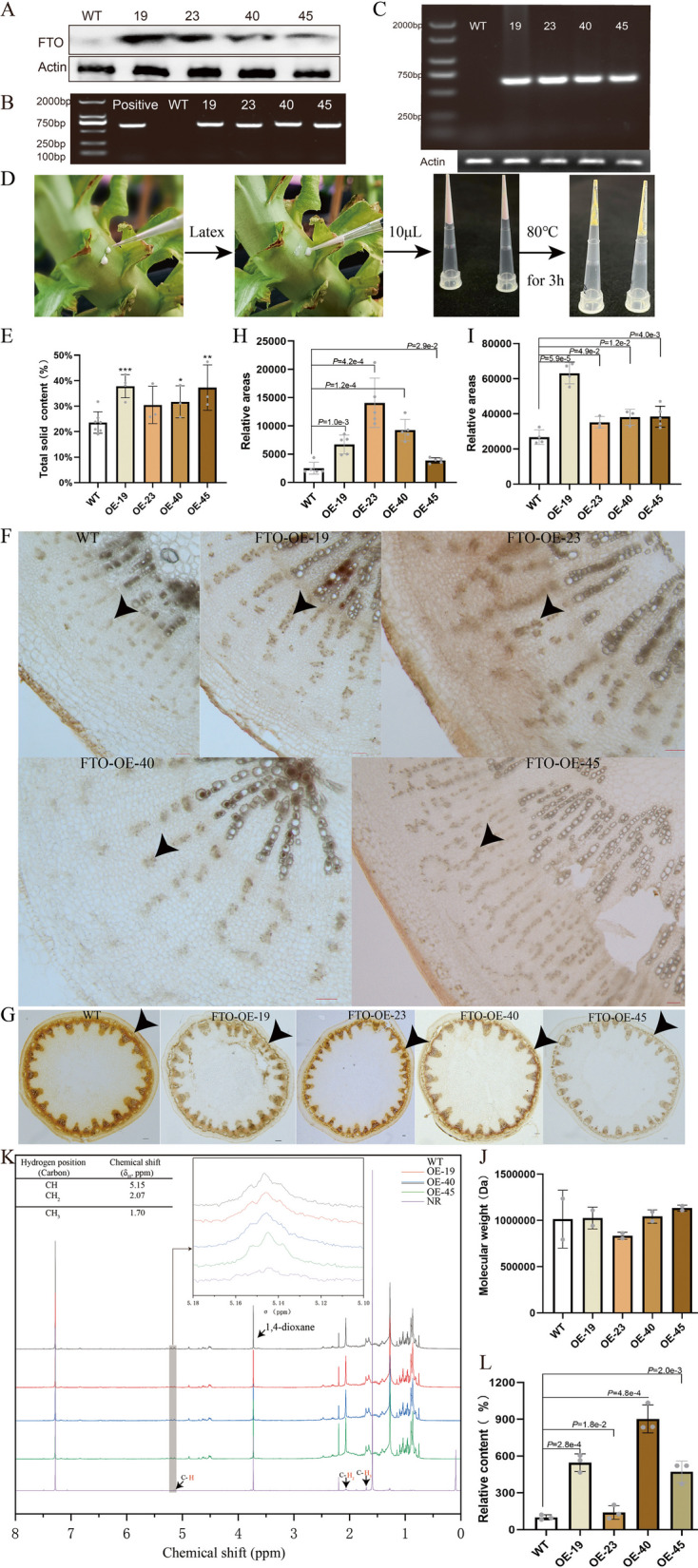
Overexpression of the demethylase *FTO* gene significantly increased laticifer index and natural rubber production in lettuce. **A** Detection of positive lines by western blotting. **B** Detection of positive lines by PCR. **C** Detection of positive lines by RT-PCR. **D** Schematic diagram of total solids content determination. **E** Determination of total solids content of latex in FTO-OE lines. Roots (**F**) and stems (**G**) were stained with iodine-bromine. Laticifer cells (indicated by black arrows) are stained dark. **H** Relative area of each column of laticifer cells of roots stained with iodine-bromine. **I** Relative area of laticifer cells of stems stained with iodine-bromine. **J** Molecular weight of NR in different FTO-OE lines by HPLC-GPC. **K** Analysis of the latex in FTO-OE lines and wild type by.^1^H-nuclear magnetic resonance (NMR). Schematic diagram of NMR. NR from the rubber tree used as positive control. **L** The relative concentration ratio of rubber hydrocarbon by NMR. * *P* ≤ 0.05, ** *P* ≤ 0.01 and *** *P* ≤ 0.001

Rubber biosynthesis begins with the sugar metabolic pathway, followed by the MVA pathway where IPP serves as the precursor for polymerization (Hu et al. 2024). Therefore, we measured the sucrose and glucose contents in the latex. The results showed that sucrose levels were significantly increased in transgenic lines (Fig. S2A), while glucose content was markedly decreased (Fig. S2B). We speculate that this phenomenon may be attributed to the substantial utilization of glucose for acetyl-CoA synthesis. To further investigate this, we determined the acetyl-CoA and isopentenyl pyrophosphate (IPP) levels in both leaves (Fig. S2C, D) and latex (Fig. S2E, F). The results demonstrated significant increases in both acetyl-CoA and IPP concentrations in transgenic lines compared to wild-type controls in these tissues.

Our study revealed that the acetyl-CoA content in latex did not exhibit a strictly linear correlation with rubber hydrocarbon content across different transgenic lines. This suggests that acetyl-CoA may not be exclusively channeled into the rubber biosynthesis pathway but could also participate in alternative metabolic processes, such as fatty acid synthesis. To test this hypothesis, we quantified pyruvate dehydrogenase (PDH) enzyme activity and fatty acid levels in the latex of various transgenic lines. The results demonstrated significantly higher enzyme activity and fatty acid content in OE-19 and OE-40 compared to OE-23 and OE-45(Fig. S2G, H). This observation supports the conclusion that acetyl-CoA is partially diverted toward fatty acid synthesis, thereby contributing to the observed reduction in acetyl-CoA concentration in latex.

Further, to detect m^6^A modifications in FTO-OE transgenic plants, we performed m^6^A-seq using MeRIP technology and conducted differential expression analysis in conjunction with RNA-seq. In this study, the m^6^A-seq results showed that the synthesis of secondary metabolites and the starch and sugar metabolism pathways undergo m^6^A demethylation in FTO-overexpressing plants (Fig. S2J), suggesting that the genes related to these pathways may be more prone to be expressed. We found a significant enrichment of m^6^A peaks for the conserved RRACH motif (R is A/G and H is A/C/U) and DRACH (D is A/G/U, R is A/G and H is A/C/U) which have been observed in other species (Fig. S3A). Figure S3B shows an example peak (*LOC111891867*) for all the tested samples. We performed GO and KEGG analyses on the differential peaks, and the results showed that an enrichment of pathways related to starch and sucrose metabolism, as well as the biosynthesis of secondary metabolites (Fig. S3C, D). Subsequently, we performed an integrative analysis of m^6^A-seq and RNA-seq data. The results of GO and KEGG analyses indicated that the differential genes were primarily enriched in starch and sucrose metabolism, biosynthesis of secondary metabolites and sulfur metabolism pathways (Fig. S3E, F). A quadrant plot analysis was conducted on a total of 2,795 significant differential genes from the two datasets. A total of 27 genes (Supplementary Material 2.) were identified significant in both m^6^A-seq and RNA-seq datasets (Fig. S3G). Among these, three genes (*LOC111890627*, *LOC111908845* and *LOC111891867*) showed upregulated expression levels concurrent with m^6^A demethylation. Although fewer genes were identified, the results are consistent with previously published findings (Fig. S2K) (Yu et al. 2021). Next, the qRT-PCR results demonstrated that the expression levels of three demethylated genes were significantly upregulated in the FTO-OE lines and were further induced by different hormones (Fig. S2I). The box plot reveals how gene expression levels are influenced by their methylation status in different functional elements. It seems that the m^6^A modification in the CDS region has a significant impact on gene expression, while the termination codon region contains the most m^6^A modifications (Fig. S3H).

Based on our findings, we have proposed a working model for the role of FTO (Fig. S2L). Overexpression of FTO leads to the demethylation of genes related to glycometabolism pathway and cell development and differentiation in lettuce. This results in increased natural rubber synthesis substrates, acetyl-CoA and IPP, as well as the proliferation of laticifer cells, culminating in the increase in NR yield in the FTO lines.

In this study, we demonstrate that the overexpression of FTO in lettuce significantly enhances the differentiation of laticifer cells and leads to a significant increased production of NR. Our findings reveal that RNA m^6^A modification plays a critical role in regulating rubber production, and its modulation provides a promising strategy for substantially elevating rubber production.

## Supplementary Information

Supplementary Material 1.

Supplementary Material 2.

## Data Availability

The raw sequence data reported in this paper have been deposited in National Genomics Data Center, China National Center for Bioinformation/Beijing Institute of Genomics, Chinese Academy of Sciences (GSA: CRA020508) that are publicly accessible at https://ngdc.cncb.ac.cn/gsa/s/yLW25Hq4.
