# Supplementary material for: Overexpression of RNA m6A demethylase FTO enhances the yield of natural rubber in lettuce
Source: Mol Hortic. 2026 Feb 6;6:11. doi: 10.1186/s43897-025-00190-y (PMC12879370; doi:10.1186/s43897-025-00190-y)
Supplement: Supplementary file 1 — Supplementary Material 1. [file 43897_2025_190_MOESM1_ESM.docx]

**Figures and figure legends
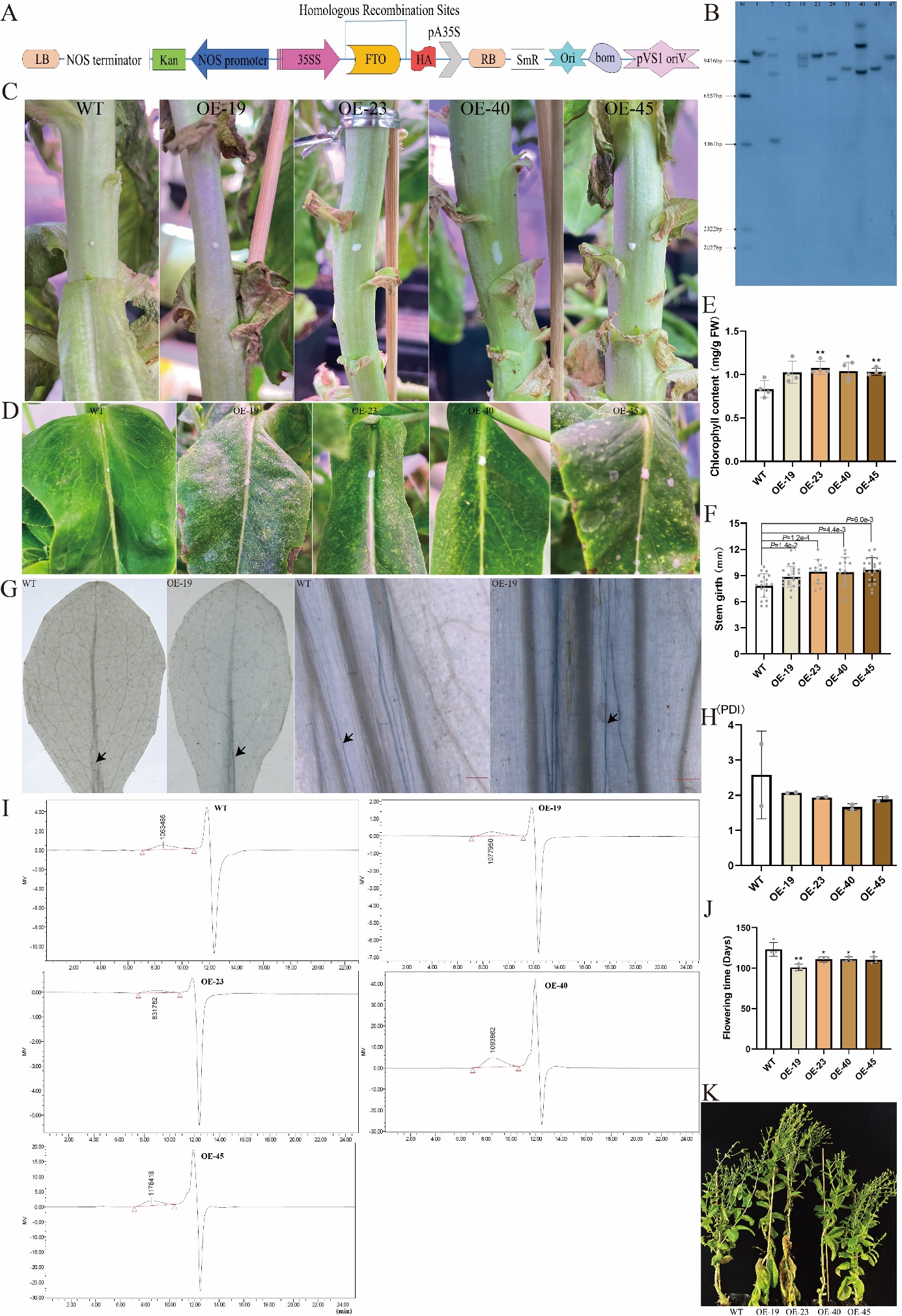
**

**Fig. S1** Phenotypic Analysis of FTO-Overexpressing Lines. **A** FTO-OE vector diagram. **B** Detection of copy number in FTO-OE lines by southern blot. **C** Needled stem showed latex outflow. **D** Needled leaves showed latex outflow. **E** Quantitative measurement of chlorophyll content in FTO-OE lines. **F** Stem girth of FTO-OE lines and wild type control. **G** Leaves stained with Sudan Black B for laticifer cells, scale bars = 500μm. **H** The polydispersity index (PDI) in the FTO-OE lines and wild type control. I High-performance liquid chromatography coupled with gel-permeation column (HPLC-GPC) chromatogram of FTO-OE lines and wild type. **J-K** Flowering time of FTO-OE lines and wild type control. * P ≤ 0.05 and ** P ≤ 0.01.


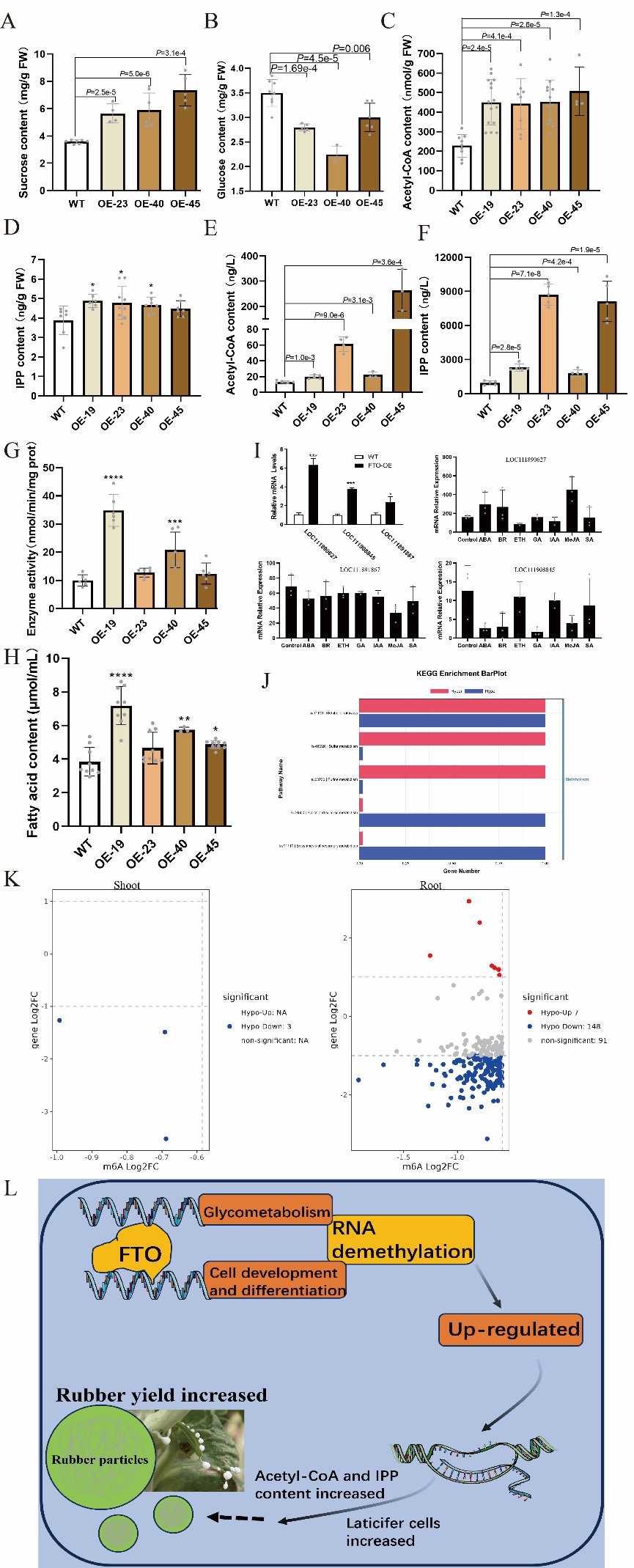


**Fig. S2** FTO promotes latex biosynthesis by regulating the expression of genes involved in sugar metabolism pathways and it’s working model. **A-B** Glucose and sucrose content of FTO-OE lines and wild type control. **C**-**D** Determination of Acetyl-CoA and IPP content in leaves. **E**-**F** Determination of Acetyl-CoA and IPP content in latex. **G** Measurement of PDH enzyme activity in latex from FTO-OE lines. **H** Measurement of fatty acid content in latex from FTO-OE lines. **I** Verification by m^6^A-IP-qPCR and the three genes response to plant hormones. Validation of Expression Levels of *LOC111890627*, *LOC111891867* and *LOC111908845* by m^6^A-IP-qPCR. Relative expression levels of each gene were measured using the 2−ΔΔCT method. Responses of the LOC111890627, LOC111891867 and LOC111908845 genes to ABA, BR, ETH, GA, IAA, MeJA and SA. Three biological replicates were used for each sample. **J** KEGG enrichment of different peaks of OE-19 vs WT. **K** Comparison of Hypo-Down and Hypo-Up gene numbers in FTO-OE lines, visualized in a scatter plot (data from published literature). **L** The working model of FTO in enhancing natural rubber yield in lettuce. FTO mediates the demethylation of genes related to glycometabolism and cell development and differentiation, promoting their expression. This leads to the accumulation of precursors for NR synthesis (Acetyl-CoA and IPP) and the proliferation of laticifer cells, finally enhancing NR yield. * P ≤ 0.05, ** P ≤ 0.01, *** P ≤ 0.001 and **** P ≤ 0.0001.

**
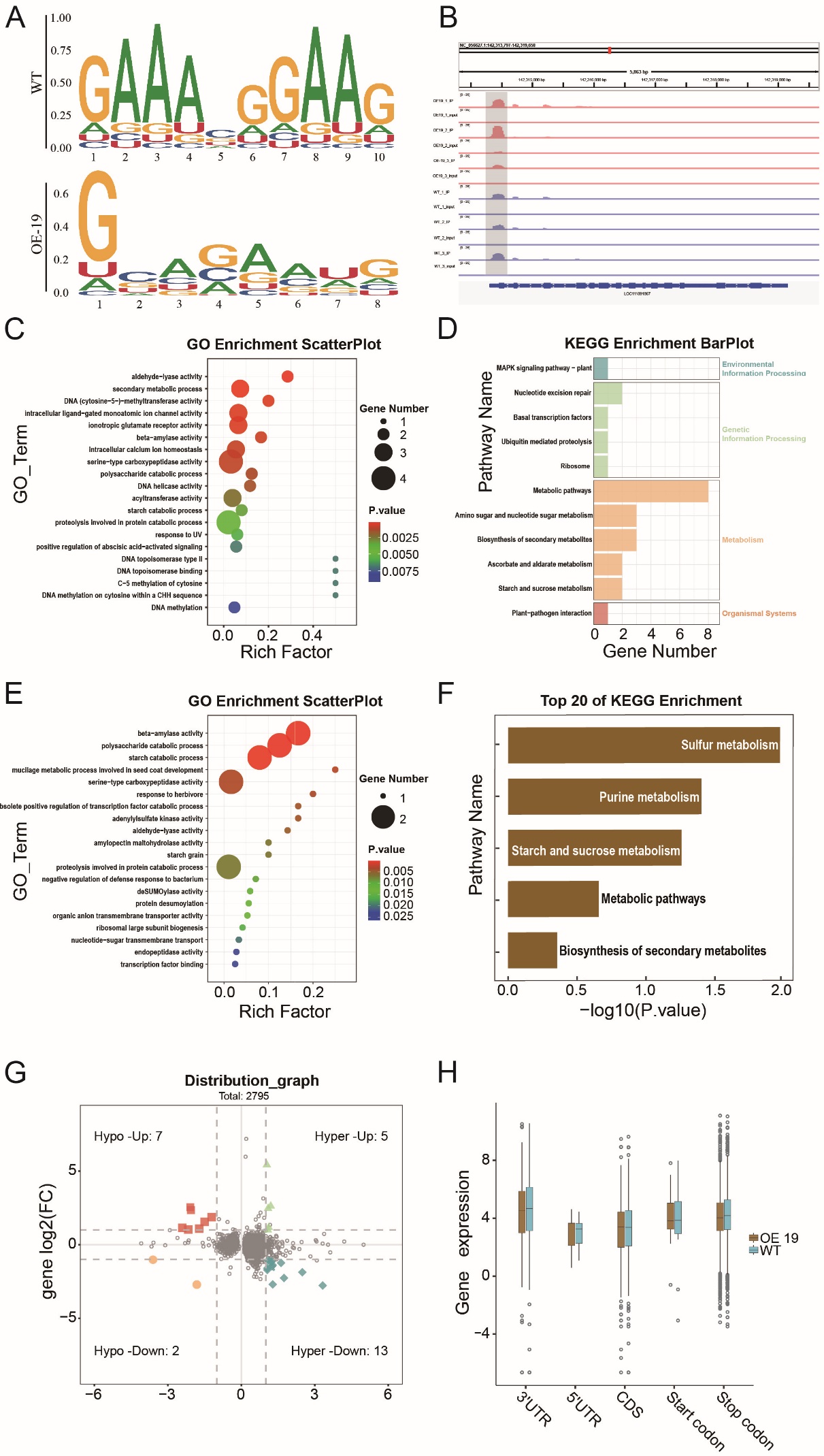
**

**Fig. S3** m^6^A-seq analysis of FTO-OE and wild-type plants. **A** The conserved RRACH sequence motif of m6A modification identified from the significant m6A peaks. **B** The transcripts of the gene (LOC111891867) as an example to show the m6A peaks (shown in dark rectangles). **C** GO analysis of differential peaks. **D** KEGG analysis of differential peaks. **E** Combined GO analysis of differentially expressed genes and differential peaks. **F** Combined KEGG analysis of differentially expressed genes and differential peaks. **G** Four-quadrant plots showing the distribution of genes with significant changes in both the m6A modification and mRNA levels. **H** The distribution of differentially expressed genes across various functional elements.

**1. Materials and methods**

**1.1.** **Plant materials and sample collection**

The DNA sequence of FTO was artificially optimized for codon usage to improve expression in plants, with its amino acid sequence consistent with NP_001073901.1. The recipient lettuce materials, *Lactuca sativa* cv Ninja, obtained from Dr. Dae-Kyun Ro were planted in a greenhouse located in Haikou, China, as well as in an agricultural ﬁeld at Huazhong Agricultural University in Wuhan, China. On the first day of flowering, latex was harvested from the stems of the transgenic lines to determine the total solid content. Three replicates of mature leaves collected for m^6^A-seq analysis were immediately placed in liquid nitrogen.

**1.2 RNA extraction and measurement of m^6^A by LC-MS/MS**

RNA from lettuce leaves was extracted using the RNAprep Pure Plant Plus Kit (DP441, Tiangen), while polyadenylated RNA (polyA-RNA) was isolated with the Dynabeads mRNA purification kit (61006, Invitrogen). A sample of 200 ng polyA-RNA underwent enzymatic treatment with 1 unit of nuclease P1 (145-07741, Wako) in 10 mM NH_4_OAc buffer (pH 5.3) for 2 hours at 42°C. Following this, MES buffer (pH 6.5, 100 mM) and 1 unit of shrimp alkaline phosphatase (M0371, New England Biolabs) were added, and the mixture was incubated at 37°C for an additional 3 hours. Simultaneously, genomic DNA was obtained from lettuce via the DNA kit (D200-100, GeneBetter); 500 ng of this DNA was processed with 2 units of DNA Degradase Plus (E2020, Zymo Research) at 37°C for 3 hours. Post digestion, the sample was centrifuged at 15,000 rpm for 30 minutes at 4°C, and 5 mL of the resulting supernatant was analyzed using LCMS/MS. Separation of nucleosides was performed using a SHIMADZU UPLC system with an Agilent ZORBAX SB-Aq column. Detection was carried out using an AB SCIEX Triple QuadTM 5500 mass spectrometer. Nucleoside modifications were quantified by measuring transitions from m/z 268.0 to 136.0 for adenine (A), and from m/z 282.0 to 150.1 for methylated adenine. Standard curves were created using pure nucleosides to determine the concentrations of A and m^6^A in the samples. The proportion of m^6^A was then expressed as a percentage of the total A. Each sample was tested in three biological replicates, with each replicate having three technical repetitions.

**1.3 m^6^A-seq assay**

A total of 25 mg of polyA-RNA was lysed with Ambion RNA Fragmentation reagent (AM8740, ThermoFisher Scientific) at 70°C for 15 min. Fragmented RNA was desalted and concentrated by ethanol precipitation. The m^6^A immunoprecipitation was performed following established methods. Fragmented polyA-RNA and m^6^A antibodies (202003, Synaptic Systems) were added to 500 mL of IP buffer (10 mM Tris-HCl pH 7.4, 300 mM NaCl and 0.1% NP-40) at 4°C and incubated overnight. An aliquot of 50 mL Protein A Dynabeads (10001D, ThermoFisher Scientific) was blocked with BSA (0.5 mg/mL); the entire reaction was carried out in IP buffer, blocked at 4°C for 2 h, then washed three times and resuspended in 100 mL of IP buffer. The blocking solution was incubated with the RNA antibody reaction solution for an additional 2 h at 4°C. The solution was then washed three times with IP buffer for 5 min each. The m^6^A-modified RNA was eluted with 6.7 mM m6A, placed on a shaker, and spun for 1 h at 4°C before final purification by ethanol precipitation. Concentrations were tested using Qubit (Thermo Fisher Scientific). Following immunoprecipitation, RNA fragments and similar quantities of fragmented polyA-RNA (as input) were used for library preparation using the NEBNext Ultra RNA Library Prep Kit for Illumina (E7530S, New England Biolabs), adhering to the guidelines provided by the manufacturer. The libraries were then sequenced using the Illumina HiSeq X Ten platform.

**1.4 m^6^A-IP-qPCR and TR-PCR**

The mRNA from each sample was cleaved with RNA Fragmentation Reagents (AM8740, ThermoFisher Scientific) to form 100 nt. The immunoprecipitation reaction was performed using EpiMark N6-Methyladenosine Enrichment Kit (E1610S, New England Biolabs). The m^6^A bound to Dynabeads Protein G (00663295, ThermoFisher Scientific), was incubated with the cleaved RNA at 4°C for 1 h. The immunoprecipitated m^6^A fragments were eluted and concentrated using Dynabeads MyOne Silane (37002D, Life Technologies). The RNA bound to the m^6^A and the input mRNA were then subjected to reverse transcription and fluorescence quantification. The experiments followed the principle of 3 biological and 3 technical replicates.

The total RNA was reverse-transcribed into cDNA using the PrimeScript 1st Strand cDNA Synthesis kit (Takara, Dalian, China). The RT-PCR amplification was performed for 28 cycles under optimized conditions. The qRT-PCR amplification was performed using SYBR® Premix Ex Taq™ II (Perfect Real Time) (Takara, Dalian, China) on the CFX96 Touch™ Real-Time PCR Detection System (Bio-Rad, Hercules, CA, USA) according to the manufacturer’s protocol. As an endogenous control, actin was used to normalize the qRT-PCR Ct values obtained, and the relative gene expression levels were calculated using the ΔΔCt method. Gene-specific primers (Supplementary Material 2.) were designed using NCBI’s Primer-BLAST (http://www.ncbi.nlm.nih.gov/tools/primer-blast/).

**1.5 Western blotting**

Total protein was extracted from lettuce leaves using the Plant Total Protein Extraction Kit (AKPR019-1, BOXBIO), and protein concentrations were determined using the BCA Protein Assay Kit. After standardizing protein concentrations, samples were mixed with protein loading buffer at a 4:1 ratio and separated by 12% SDS-PAGE. Proteins in the gel were then transferred to a PVDF membrane and washed three times with 1× TBST buffer, each for 5 minutes. The membrane was blocked with 5% skimmed milk at room temperature for 3 hours, followed by three washes with 1× TBST buffer, each lasting 5 minutes. Anti-FTO polyclonal antiserum was added, and the membrane was incubated at room temperature for 3 hours, followed by three washes with 1× TBST buffer, each for 5 minutes. Finally, the blot was incubated with secondary antirabbit IgG and visualized using Clarity Western ECL Substrate (BIORAD).

**1.6** **Analysis of agronomic and physiological traits**

**Flowering time.** Flowering data calculated from the sowing date were recorded when the first plant bloomed.

**Needled stem showed latex outflow.** Select the plants in the peak flowering stage, take a distance of 15 cm from the soil surface and pierce the main stem with a 1mL syringe needle with a diameter of 0.45 mm, and the depth is fixed at 2mm.

**Determination of total solids content.** The 20μL white pipette head was weighed and recorded with an analytical balance, the plants at the peak flowering stage were selected, the rubber was tapped and the latex was sucked with a 20μL pipette head and the fresh weight was weighed. It was placed in an 80°C oven for drying for 3h and weighed.

**Chlorophyll determination.** Approximately 100 mg of leaves from each sample were weighed into a 2 mL centrifuge tube, with each sample divided into three aliquots to provide three technical replicates. To each tube, 1.5 mL of extraction buffer was added. The extraction was performed in the dark at 4°C for 12 hours. The absorbance of the chlorophyll extract was measured at wavelengths of 645 nm and 663 nm using a UV-Vis spectrophotometer DU640, with the extraction buffer serving as the blank.

**Laticifer cell staining.** We employed two different staining methods to visualize the laticifer cells in the plants. Stems and roots were collected from lettuce plants at the flowering stage. The stems were cut at a point 15 cm above the soil surface and submerged in 80% ethanol. The roots were thoroughly cleaned with water to remove any soil and fine roots before the main root sections were fully immersed in 80% ethanol. Sections were then prepared from portions of the stems and roots with similar diameters for consistent analysis. Stem and roots were stained with iodine-bromine solution (5 g iodine, 0.4 mL bromine, and 100 mL glacial acetic acid). Fresh samples of stems and roots were fixed in 80% (v/v) ethanol for 24 hours (Castelblanque et al., 2018). These samples were then sliced using a vibratome (Leica, Germany) with the following parameters: speed = 1.00 mm/s, amplitude = 1.0 mm, and thickness = 100 μm. The samples were then immersed in 95% ethanol for 30 minutes, followed by dehydration in anhydrous ethanol over 2-3 cycles, each lasting 30 minutes. Next, the samples were immersed in ice-cold acetic acid for 10 minutes, and then treated in iodine-bromine solution at 60°C for 2-4 hours. After that, the samples were washed with ice-cold acetic acid three times, each for 10 minutes. Finally, ice-cold acetic acid was added for washing overnight. Images were captured using a light microscope SZX16 (Olympus, Japan)(Chao et al., 2023; Zhang et al., 2015).

Leaves were stained with Sudan Black B for laticifer observation. Take the seedlings that have grown for 2 weeks and incubated the whole seedlings in FAA overnight, then rinsed three times with 70% ethanol, and subsequently stained with Sudan Black B at room temperature for 12 hours. After staining, the leaves were washed three times with 70% ethanol, followed by three water rinses. They were then immersed in 2.5 M sodium hydroxide for 3 hours to remove the mesophyll.

**The isopentenyl pyrophosphate (IPP) content determination.** Using 100 mg of lettuce leaves at the rosette stage, total protein was extracted using a protein extraction kit, latex was collected from the lettuce flowering stage, and C-serum was isolated. Finally, the assay is determined using a commercial kit from Shanghai Kexing Trading Co., Ltd.

**The acetyl-CoA content was determination.** The methods of obtaining total protein of lettuce leaves and latex C-serum were consistent with those in the IPP content determination experiment. Finally, the assay is determined using a commercial kit from Grace Biologics, Inc.

**1.7 NMR and HPLC-GPC analysis of NR**

For HPLC-GPC (Waters 1515), take 50 mg of lettuce latex from flowering lettuce and place it in a brown sampling bottle with 1 mL of acetone. The vial was gently shaken to ensure that the latex was fully immersed in the acetone and stood for 24 hours. The acetone solution was then discarded, and the vial was placed in a fume hood to evaporate any residual acetone. Subsequently, 800-1000 μL of tetrahydrofuran (THF, chromatography grade) was added, and the mixture was allowed to stand for 3 days. The THF was filtered using disposable filters and transferred to a new sampling vial for testing. Where the solution was viscous, additional THF was added to dilute it and prevent filter column clogging. Where the solution was turbid, a new filter was used for re-filtration. The gel permeation chromatography system was used with a WAT054460 column (7.8 × 300 mm). THF was used as the mobile phase at a flow rate of 1 mL/min with isocratic elution. A 30 μL injection was made, with an injection time of 25 minutes and internal and external detector temperatures set to 40°C. To determine the weight average molecular weight (Mw), polystyrene (PS) standard (Waters) dissolved in THF was used. Gel permeation chromatography was performed after complete dissolution, and a standard curve was established using Breeze2 software (Waters, USA) which was also employed to integrate the signal peaks of the chromatograms. The weight average Mw and number average molecular weight (Mn) of the NR samples were calculated using the PS standard curve. The polydispersity index (PDI) was computed by dividing Mw by Mn.

Quantitative NMR utilizing 1,4-dioxane as an internal standard was employed to determine the concentration of NR in the lettuce latex samples. Each weighed dry rubber simple (about 30 mg) was processed following the steps of Kwon et al. (Kwon et al., 2023). There were 3 biological replicates for each sample. The latex from wild type lettuce and NR from *Hevea brasiliensis* rubber trees served as controls.

**1.8 Statistical analysis**

The means ± standard errors (SE) were calculated with all statistical analyses performed using SPSS v17. Significant differences between the means of the treatment groups were determined by the Student's *t*-test at P < 0.05.

Castelblanque, L., Balaguer, B., Marti, C., Orozco, M. and Vera, P. (2018) LOL2 and LOL5 loci control latex production by laticifer cells in Euphorbia lathyris. *New Phytol* **219**, 1467-1479.

Chao, J.Q., Wu, S.H., Shi, M.J., Xu, X., Gao, Q., Du, H.L., Gao, B., Guo, D., Yang, S.G., Zhang, S.X., Li, Y., Fan, X.L., Hai, C.Y., Kou, L.Q., Zhang, J., Wang, Z.W., Li, Y., Xue, W.B., Xu, J., Deng, X.M., Huang, X., Gao, X.S., Zhang, X.F., Hu, Y.S., Zeng, X., Li, W.G., Zhang, L.S., Peng, S.Q., Wu, J.L., Hao, B.Z., Wang, X.C., Yu, H., Li, J.Y., Liang, C.Z. and Tian, W.M. (2023) Genomic insight into domestication of rubber tree. *Nature Communications* **14**.

Kwon, M., Hodgins, C.L., Salama, E.M., Dias, K.R., Parikh, A., Mackey, A.V., Catenza, K.F., Vederas, J.C. and Ro, D.K. (2023) New insights into natural rubber biosynthesis from rubber-deficient lettuce mutants expressing goldenrod or guayule cis-prenyltransferase. *New Phytologist* **239**, 1098-1111.

Zhang, S.X., Wu, S.H., Chen, Y.Y. and Tian, W.M. (2015) Analysis of Differentially Expressed Genes Associated with Coronatine-Induced Laticifer Differentiation in the Rubber Tree by Subtractive Hybridization Suppression. *PLoS One* **10**, e0132070.
