# Supplementary material for: Overexpression of RNA m6A demethylase FTO enhances the yield of natural rubber in lettuce
Source: Mol Hortic. 2026 Feb 6;6:11. doi: 10.1186/s43897-025-00190-y (PMC12879370; doi:10.1186/s43897-025-00190-y)
Supplement: Supplementary file 2 — Supplementary Material 2. [file 43897_2025_190_MOESM2_ESM.docx]

1. **Primers used in this study.**

| **LOC111890627** | 1F: CATGCTGGCGAACTATGTGC |
| --- | --- |
|  | 1R: ACGTCACACATCACCCCATC |
| **LOC111908845** | 2F: ATGCAGCGGTTGGTGATACA |
|  | 2R: TGCAAACCAAAGCATCCTGC |
| **LOC111891867** | 3F: ACCCTCTCATGCTTTGGCTC |
|  | 3R: GCTGCCTTTGTCCAACTGTG |
| F_fto_ | CACTTGGCTCCCTTATCTGAC |
| R_fto_ | CGTTGTATGCTGCTCTGCTCTTA |

1. **27 genes identified in the quadrant plot analysis.**

gene_name Description

LOC111885984 stress-response A/B barrel domain-containing protein At5g22580 [Lactuca sativa]

LOC111888908 uncharacterized protein LOC111888908 isoform X1 [Lactuca sativa];uncharacterized protein LOC111888908 isoform X2 [Lactuca sativa]

LOC111882527 serine carboxypeptidase-like [Lactuca sativa]

LOC111900212 adenylyl-sulfate kinase 3-like [Lactuca sativa]

LOC111903239 protein HOTHEAD [Lactuca sativa]

LOC111881582 none

LOC111905211 cytoplasmic 60S subunit biogenesis factor REI1 homolog 1-like [Lactuca sativa]

LOC111894411 protein DETOXIFICATION 12-like [Lactuca sativa]

LOC111877948 snakin-2-like isoform X1 [Lactuca sativa]

LOC122198033 protein ALP1-like [Lactuca sativa]

LOC111893365 interactor of constitutive active ROPs 4-like [Lactuca sativa]

LOC111889410 F-box protein At2g26850-like [Lactuca sativa]

LOC111892810 hypothetical protein LSAT_8X101260 [Lactuca sativa]

LOC111907104 inactive beta-amylase 9-like [Lactuca sativa]

LOC111891867 serine carboxypeptidase-like 13 [Lactuca sativa]

LOC111885055 beta-amyrin 28-oxidase-like [Lactuca sativa]

LOC111891521 E3 ubiquitin-protein ligase MIEL1-like [Lactuca sativa]

LOC111893374 hypothetical protein LSAT_5X124321 [Lactuca sativa]

LOC111892608 none

LOC111889859 hypothetical protein LSAT_9X81600 [Lactuca sativa]

LOC111898783 putative retrovirus-related Pol polyprotein from transposon TNT 1-94 [Helianthus annuus]

LOC111908845 glutamate receptor 2.9-like [Lactuca sativa]

LOC111901011 chitin-inducible gibberellin-responsive protein 1-like [Lactuca sativa]

LOC111920117 probable sugar phosphate/phosphate translocator At3g10290 [Lactuca sativa]

LOC111917061 uncharacterized protein LOC111917061 isoform X1 [Lactuca sativa];uncharacterized protein LOC111917061 isoform X2 [Lactuca sativa]

LOC111890627 beta-amylase isoform X2 [Lactuca sativa];beta-amylase isoform X1 [Lactuca sativa]

LOC111898223 protein LURP-one-related 10-like [Lactuca sativa]
